# Morphology and fine organization of the midgut of *Gampsocleis gratiosa* (Orthoptera: Tettigoniidae)

**DOI:** 10.1371/journal.pone.0200405

**Published:** 2018-07-09

**Authors:** Ke Li, Jin-Hui Zhang, Yu-Jing Yang, Wei Han, Huan Yin

**Affiliations:** 1 College of Life Sciences, Shanxi Normal University, Linfen, Shanxi, China; 2 Modern College of Humanities and Sciences, Shanxi Normal University, Linfen, Shanxi, China; Biocenter, Universität Würzburg, GERMANY

## Abstract

The morphology and ultrastructure of the midgut of *Gampsocleis gratiosa* (Orthoptera: Tettigoniidae) was examined by light and electron microscopy. The midgut consists of two bulbous gastric caeca and a tubular ventriculus. The general organization of the gastric caeca is similar to that of the ventriculus. They are composed of a peritrophic membrane, an epithelium, a basal lamina and muscle layer from the inside to outside. Three types of cells were identified: regenerative, principal, and endocrine. Regenerative cells occur in groups (called nidi) at the base of principal cells. Principal cells grow from regenerative cells. Rare endocrine cells are scattered throughout the epithelium. Principal cells exhibit intense secretory activity, and regional differences in their ultrastructure were observed along the entire midgut. The microvilli are longer than those in any other region in the posterior region of the midgut. Lysosomes, multivesicular bodies (MVBs), autophagosomes, abundant Golgi apparatuses and lipid droplets primarily occur in the gastric caeca. Three pathways of secretion (merocrine, apocrine and holocrine) occur within the midgut epithelium, and a distinctive type of apocrine bleb was found in the gastric caeca. Therefore, these gastric caeca may be evolving toward a special type of gland.

## Introduction

The entire alimentary canal of insects traverses the body from mouth to anus and is generally separated into three major parts, the foregut, midgut, and hindgut, according to embryological origin [1, 2]. The origin of the foregut and hindgut is the ectoderm, whereas the midgut derives from the endoderm. The foregut is primarily involved in receiving, transporting and initially digesting food; the hindgut has important roles in many physical processes of insects, and the midgut serves as the primary section for enzyme production and secretion, food digestion, and nutrient absorption [2, 3].

In most insects, the midgut consists of two parts, the gastric caeca and the ventriculus. Gastric caeca are midgut projections that are an evolutionary consequence of the feeding habits of insects. These projections vary among taxa in shape, number, size and site from which they arise from the midgut [2]. In addition, midgut epithelial cells show regional ultrastructural differentiation, a characteristic indicating that cells from various regions have different functions [4–7].

The structure and morphology of the alimentary canal traces the evolution of the digestive system in Orthoptera [8, 9], an order that is divided into two major suborders: Caelifera and Ensifera. The latter includes the families Gryllidae and Tettigoniidae. The digestive system of members of Caelifera is fairly well known [10–12], whereas relatively little information is available regarding ultrastructural organization in Tettigoniidae. Therefore, we examined the morphology and structure of the midgut of *Gampsocleis gratiosa* (Orthoptera: Tettigoniidae) using microscopy techniques to extend our knowledge and to provide basic data on the functions and evolution of the insect digestive system.

## Materials and methods

Healthy *G*. *gratiosa* adults were obtained from the Huo Mountains in Linfen, Shanxi Province, China. The insects were reared in the laboratory at room temperature (25°C) under a natural photoperiod and fed carrots.

Twenty specimens were anaesthetized under light CO_2_, and the entire alimentary canals were dissected in a physiological saline solution containing (in mM) 130 NaCl, 5 KCl, 2 MgCl_2_, and 2 CaCl_2_ at pH = 7.4. The morphology of the midgut was identified under a stereomicroscope, and the midgut was sectioned into four parts: the gastric caeca, anterior ventriculus, middle ventriculus and posterior ventriculus. For histological investigations, samples were fixed in Bouin’s liquid for 4 h at 4°C, dehydrated in a graded series of alcohol for 10 min each, and embedded in paraffin. Sections (7 μm thick) were stained with hematoxylin-eosin.

For the scanning electron microscope (SEM), samples were fixed in 2.5% glutaraldehyde for 24 h at 4°C. After rinsing 3 times with phosphate-buffered saline (PBS), the samples were dehydrated in a graded series of ethanol that transitioned to tertiary butyl alcohol, followed by freeze-drying using a FD-1-50 vacuum freeze dryer (Boyikang Laboratory Instruments Co., Ltd, Beijing, China) at -20°C for 12 hours. Thereafter, the samples were mounted on stubs, gold coated and observed under a JSM-7500F scanning electron microscope.

Preparation of samples for transmission electron microscopy (TEM) followed that of Li (2016) [13]. Ultrathin sections (60 nm) were cut by using a glass knife on Reichert and stained with uranyl acetate and lead citrate, and photographed using an H-7650 transmission electron microscope.

### Ethics statement

No specific permits were required for the described field studies: a) no specific permissions were required for these locations/activities; b) locations were not privately-owned or protected; and c) the field studies did not involve endangered or protected species.

## Results

**Fig 1:**
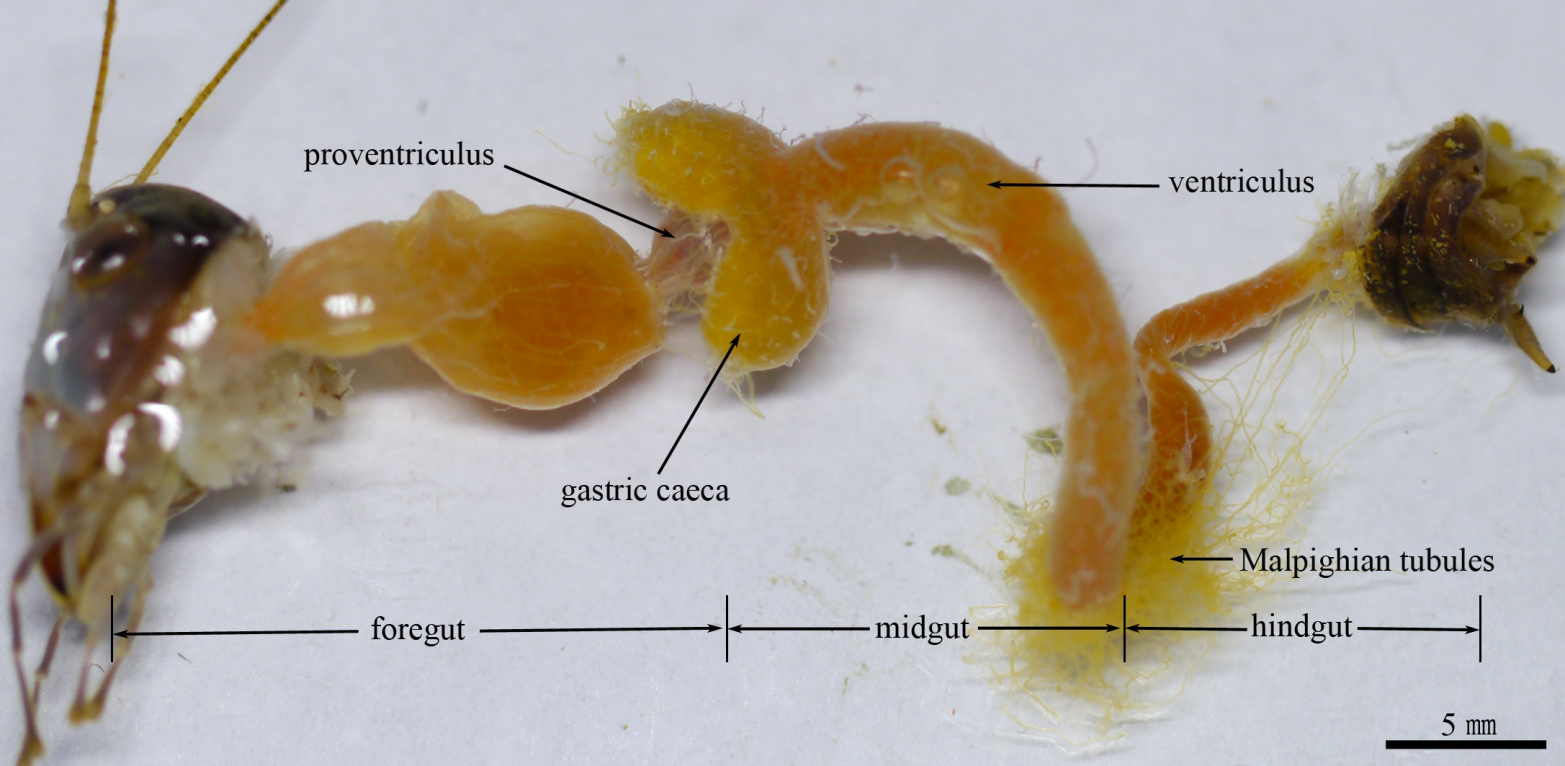
Anatomical view of the alimentary canal of *G*. *gratiosa*. Scale bar = 5 mm.

The alimentary canal of *G*. *gratiosa* is divided into the foregut, midgut, and hindgut (Fig 1). The midgut consists of two large gastric caeca attached at the anterior end and a tubular ventriculus (Fig 1). The distal ends of both bulbous gastric caeca are directed forward and embrace the proventriculus (Fig 1). The dorsal and ventral caeca are similar in size at approximately 6 mm in length and 3.1 mm in width at the widest point. A tubular ventriculus (approx. 2.5 mm in length) almost uniform in diameter (approx. 2 mm) extends to the hindgut.

**Fig 2:**
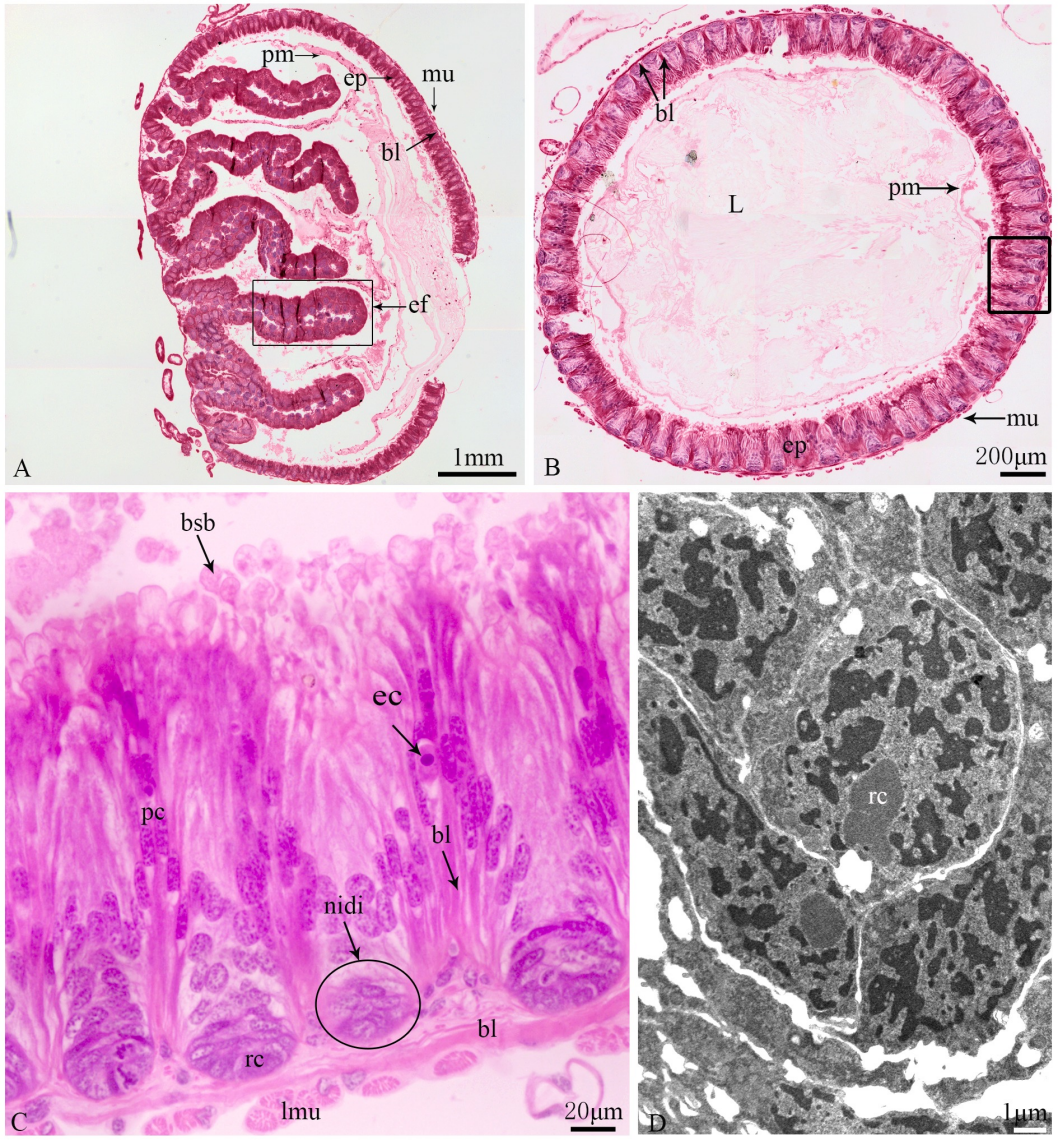
General organization of the midgut in *G*. *gratiosa*. (A) Longitudinal section of the gastric caeca. (B) Transverse section of the ventriculus. (C) The region delimited by the rectangle in (B). (D) Regenerative cells under TEM. pm: peritrophic membrane; ep: epithelium; bl: basal lamina; mu: muscle layer; ef: epithelial fold; rc: regenerative cell; pc: principal cell; ec: endocrine cell; lmu: longitudinal muscle; L: lumen. Scale bars: (A) = 1 mm; (B) = 200 μm; (C) = 20 μm; (D) = 1 μm.

The general organization of the gastric caeca and the ventriculus is similar. From the inner to the outer layer, the peritrophic membrane, epithelium, basal lamina and muscle layer constitute the midgut (Fig 2A–2C). The peritrophic membrane was observed to envelop the food bolus in the entire midgut lumen (Fig 2A and 2B), and a space was observed between the peritrophic membrane and the epithelium (Fig 2A and 2B). Seven epithelial folds (Fig 2A) of different lengths pointing toward the lumens of the gastric caeca were only found in the apical region of the gastric caecum and not in other regions of the midgut.

**Fig 3:**
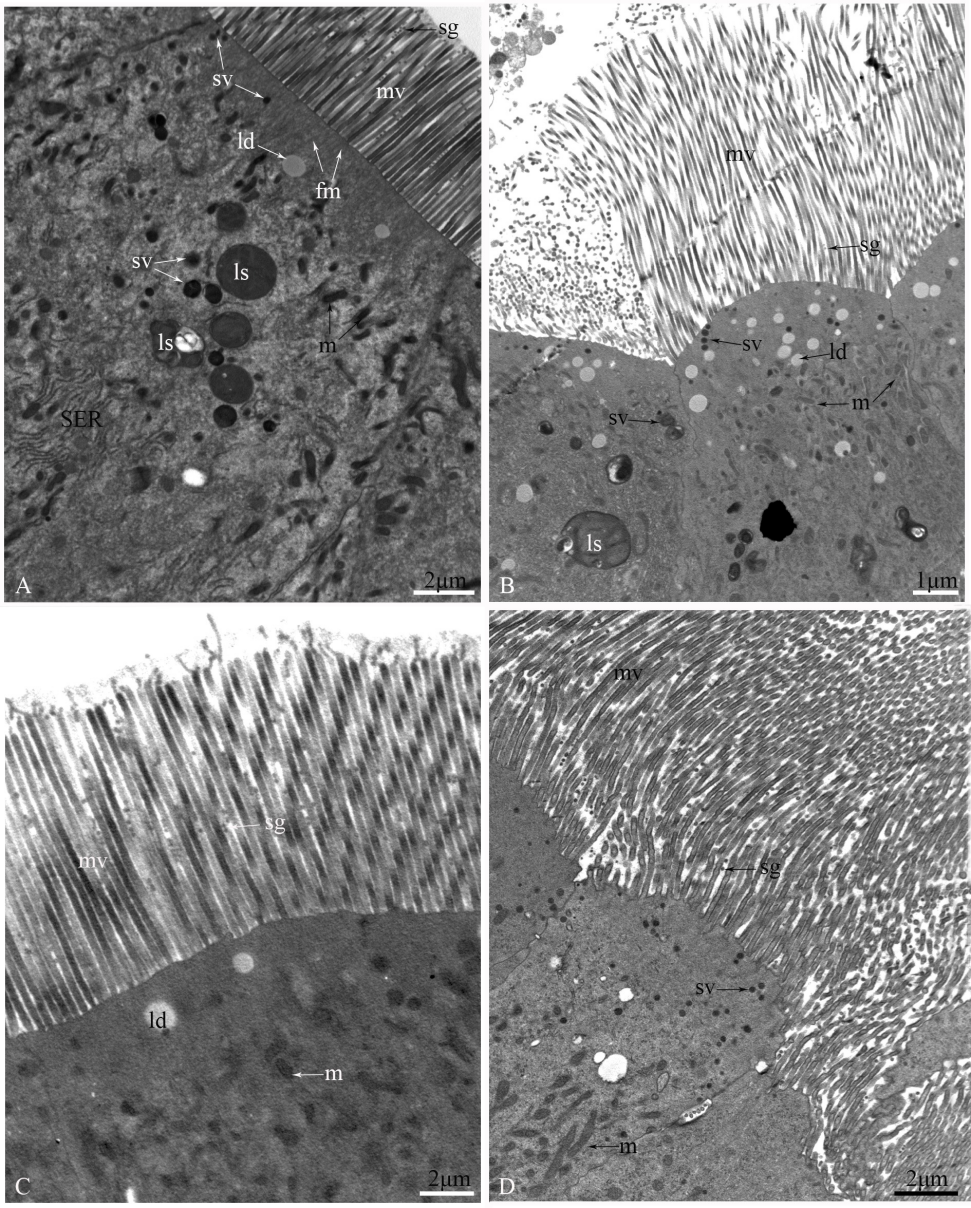
The apical region of principal cells from four parts of the midgut of *G*. *gratiosa*, as observed by TEM. (A) The gastric caeca. (B) The anterior ventriculus. (C) The middle ventriculus. (D) The posterior ventriculus. sv: secretory vesicle; mv: microvilli; sg: secretory granule; ld: lipid droplet; fm: filaments; ls: lysosomes; m: mitochondria; SER: smooth endoplasmic reticulum. Scale bars: (A) = 2 μm; (B) = 1 μm; (C) = 2 μm; (D) = 2 μm.

**Fig 4:**
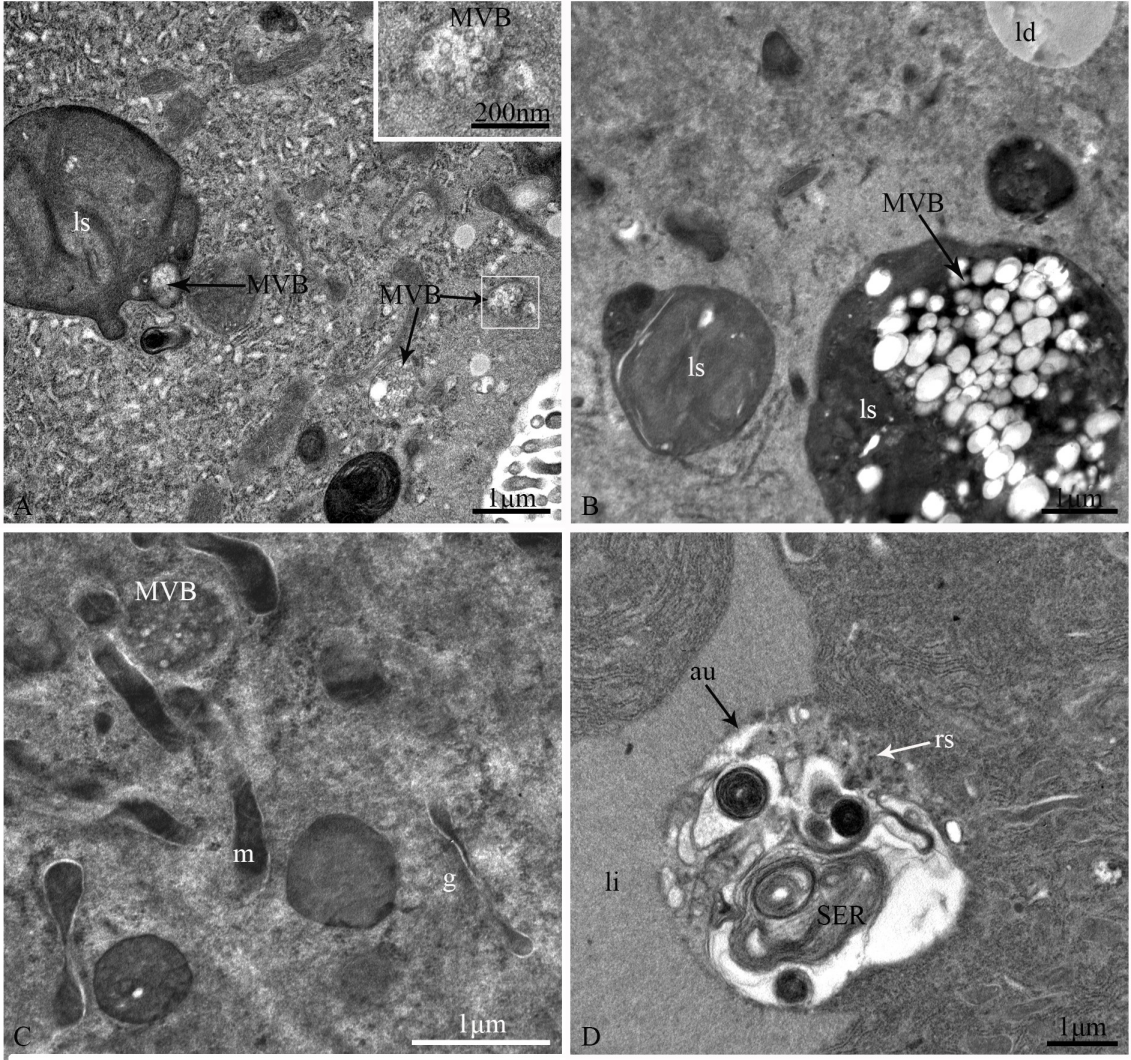
TEM photographs of various-sized lysosomes, multivesicular bodies (MVBs), autophagosomes and a few lipid droplets. (A) MVB endocytosed by a lysosome. The inset shows the dilation of the MVB in the white rectangle. The MVBs contain dense material, similar to a secretory vesicle. (B) Lipid droplets hydrolyzed by a lysosome. (C) The MVBs contain dense material, which appears identical to the contents of secretory vesicles. (D) An autophagosome containing cytosolic components in the middle zone of a principal cell. ls: lysosome; MVB: multivesicular body; ld: lipid droplet; m: mitochondria; g: Golgi apparatuses; li: lipid; au: autophagosome. Scale bars: (A) = 1 um; (A, inset) = 200 nm; (B-D) = 1 μm.

**Fig 5:**
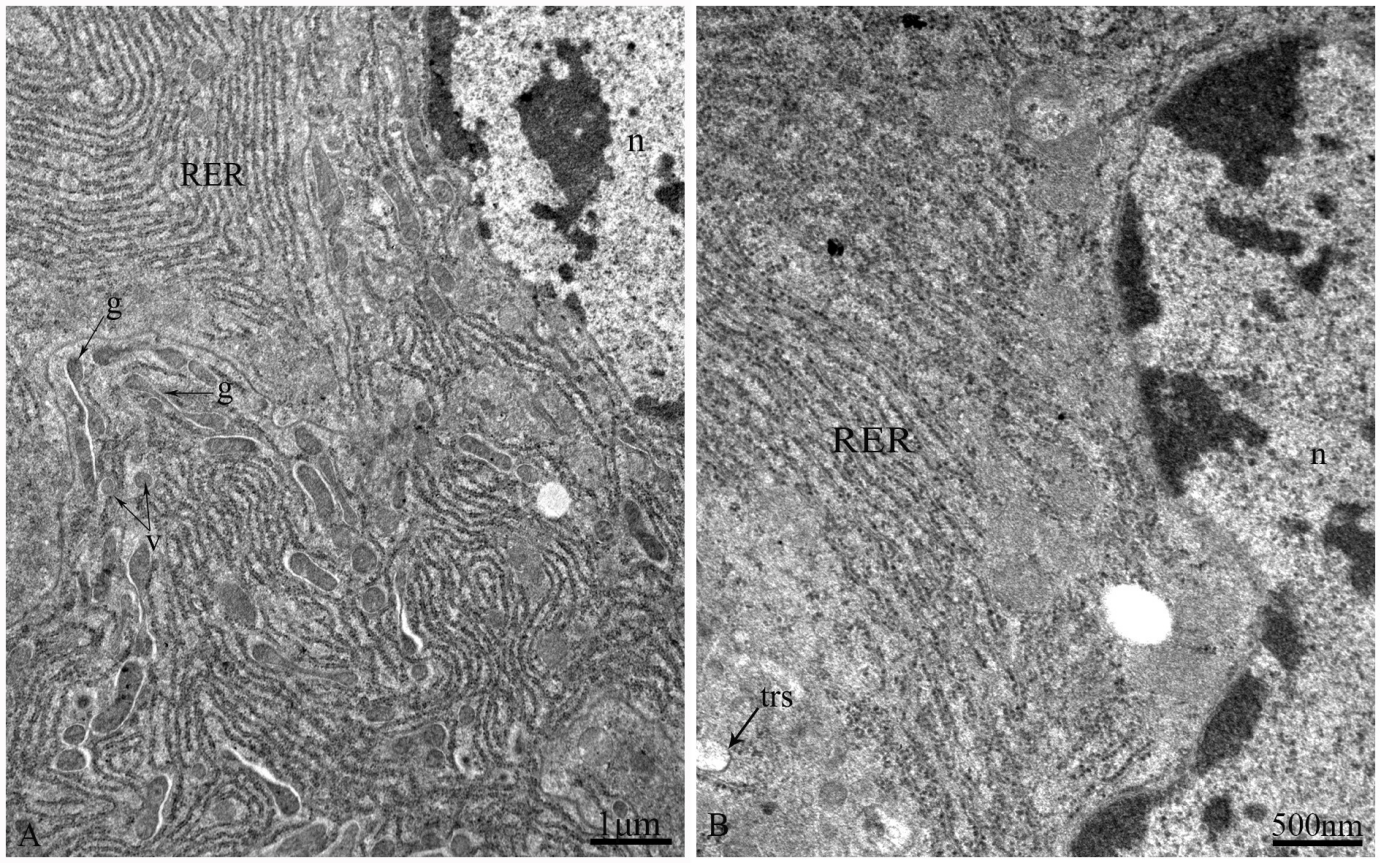
The middle zone of principal cells of the midgut of *G*. *gratiosa*, TEM. (A) The gastric caeca. (B) The ventriculus. n: nucleus; RER: rough endoplasmic reticulum; g: Golgi apparatuses; v: vesicle; trs: tracheoles. Scale bars: (A) = 1 μm and (B) = 500 nm.

**Fig 6:**
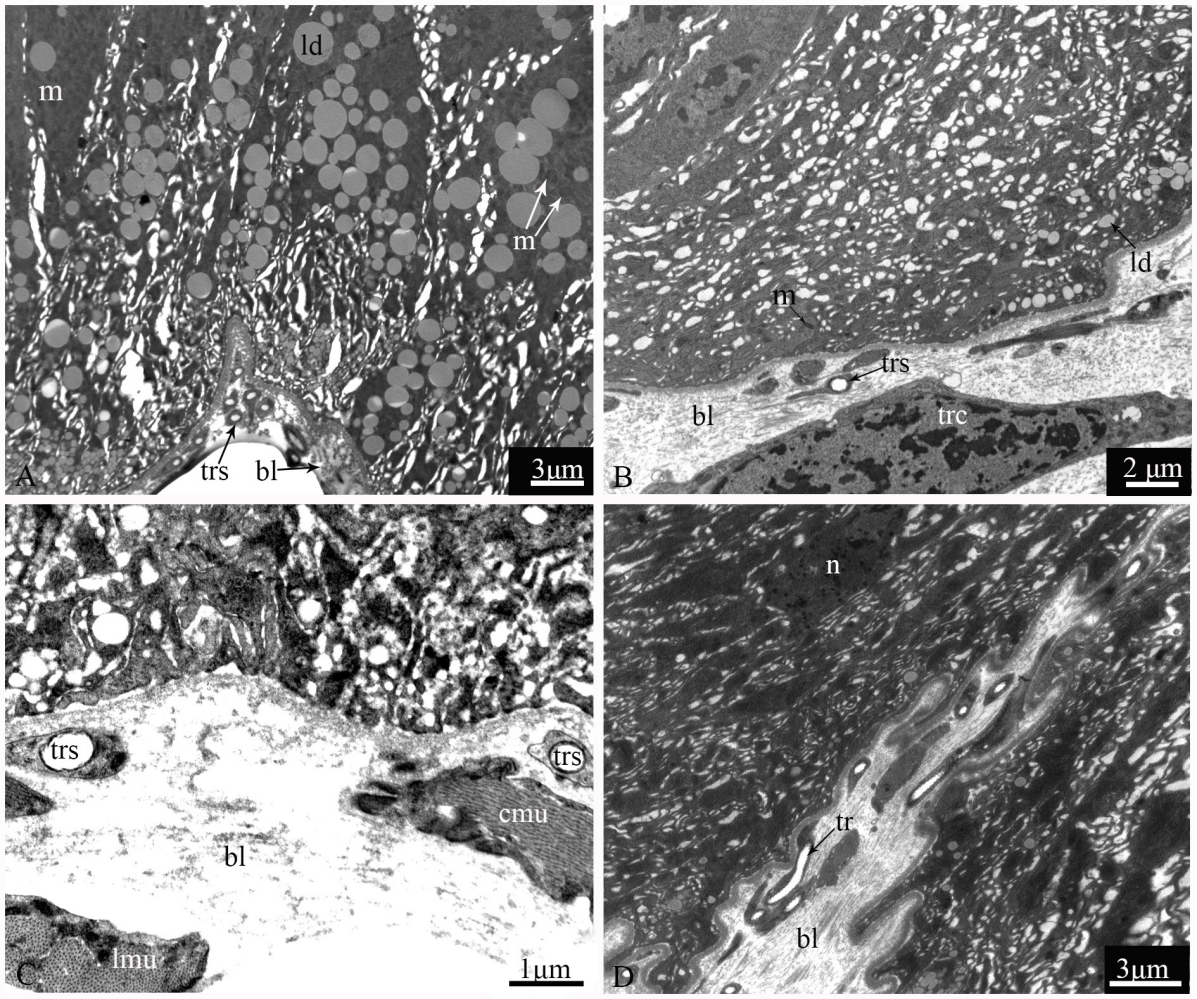
The basal region of principal cells from four parts of the midgut of *G*. *gratiosa*, TEM. (A) The gastric caeca. (B) The anterior ventriculus. (C) The middle ventriculus. (D) The posterior ventriculus. ld: lipid droplet; trs: tracheoles; bl: basal lamina; m: mitochondria; trc: tracheolar cell; cmu: circular muscle; lmu: longitudinal muscle; tr: tracheas; n: nucleus. Scale bars: (A and D) = 3 μm; (B) = 2 μm; (C) = 1 μm.

**Fig 7:**
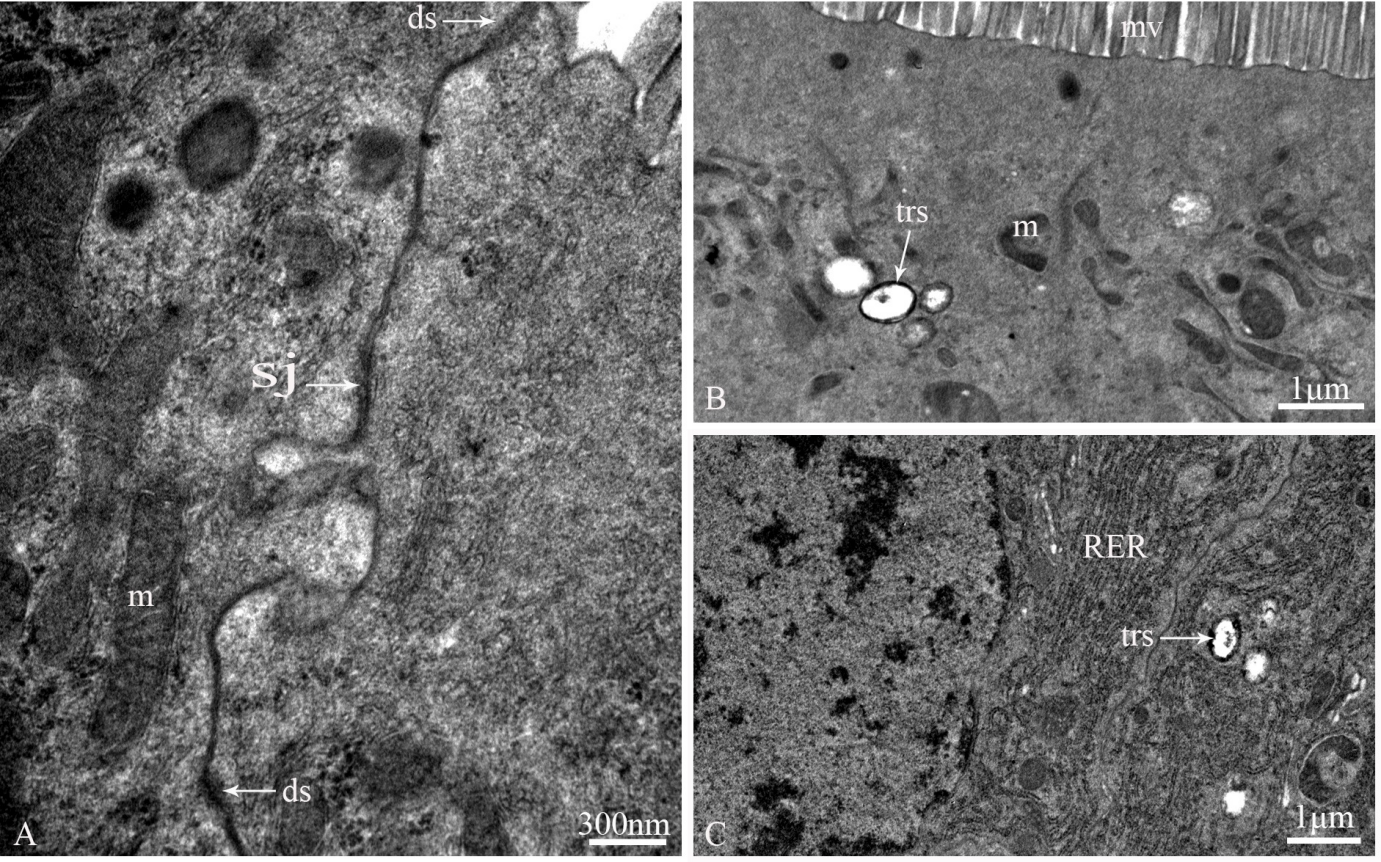
Cell junctions of principal cells and the distribution of tracheoles in the midgut of *G*. *gratiosa*, TEM. (A) Desmosomes and septate junctions with deep interdigitations of an adjacent cell. (B) Tracheoles in the apical region of a principal cell. (C) Tracheoles in the middle region of a principal cell. ds: desmosomes; sj: septate junction; m: mitochondria; trs: tracheoles; mv: microvilli; RER: rough endoplasmic reticulum. Scale bars: (A) = 300 nm and (B and C) = 1 μm.

The epithelium is composed of three cell types: regenerative cells, principal cells and endocrine cells (Fig 2C). These cells adhere to the basal lamina (Fig 2C), which extends serially from the base to the apex of the epithelium, with numerous nidi developed (Fig 2A–2C). Regenerative cells occur in groups at the bottom of the nidi (Fig 2C and 2D) and are characterized by a large nucleus with heterochromatin as well as scant cytoplasm and few organelles (Fig 2C and 2D). Principal cells are continually replaced from regenerative cells. According to the ultrastructural features of principal cells by TEM, these cells are involved in support, secretion and absorption functions. The apical membrane of principal cells forms numerous microvilli measuring approximately 4.8 μm in length in the caecum, 6.2 μm in the anterior ventriculus, 10.5 μm in the middle ventriculus, and approximately 12.6 μm in the posterior ventriculus (Fig 3). Many dark secretory granules can be observed among the microvilli (Fig 3). In the middle region of principal cells is an elliptical nucleus with heterochromatin and euchromatin. Three regions of cytoplasm are found between the microvilli and nucleus. The apical zone contains filaments (Fig 3A). The middle zone contains abundant polymorphous mitochondria, a small amount of smooth endoplasmic reticulum (SER) and secretory vesicles that vary in electron-density and size (Fig 3). Various-sized lysosomes, multivesicular bodies (MVBs) and a few lipid droplets were primarily observed in this zone of the caeca (Figs 3A, 3B and 4). Near the nucleus, the basal zone is richly supplied with rough endoplasmic reticulum (RER) (Fig 5) occurring as groups of elongated cisternae usually arranged in parallel arrays or in tight whorls. Golgi apparatuses are plentiful among the RER of the caeca and are composed of a single flattened membrane-bounded cisterna and associated vesicles (Fig 5A). A few autophagosomes were also observed in this zone (Fig 4D). Extensive infoldings formed by the basal plasma membrane have an irregular branch-like structure (Fig 6), which together constitute a basal labyrinth system of channels that could reach almost to the central region of the cells (Fig 6D). Lipid droplets of variable size occur among the basal labyrinth and are particularly abundant in that of the caeca (Fig 6A). Mitochondria are abundant and associated with basal infoldings in the basal area of the principal cells (Fig 6). The lateral continuous plasma membranes of adjacent cells form desmosomes and septate junctions with deep interdigitations (Fig 7A).

The basal lamina is an electron-lucent layer composed of banded fibrils (Fig 6). The muscle layer consists of an inner substratum of circular muscle and an outer sheath of longitudinal muscle (Fig 6C), which are embedded inside and out by the basal lamina. Tracheolar cells were also observed here (Fig 6B). Tracheas ramify into the basal lamina and reach the tracheolar cells, which form multiple tracheoles that are less than 1 μm in diameter and penetrate each cell of the body (Fig 7B and 7C).

**Fig 8:**
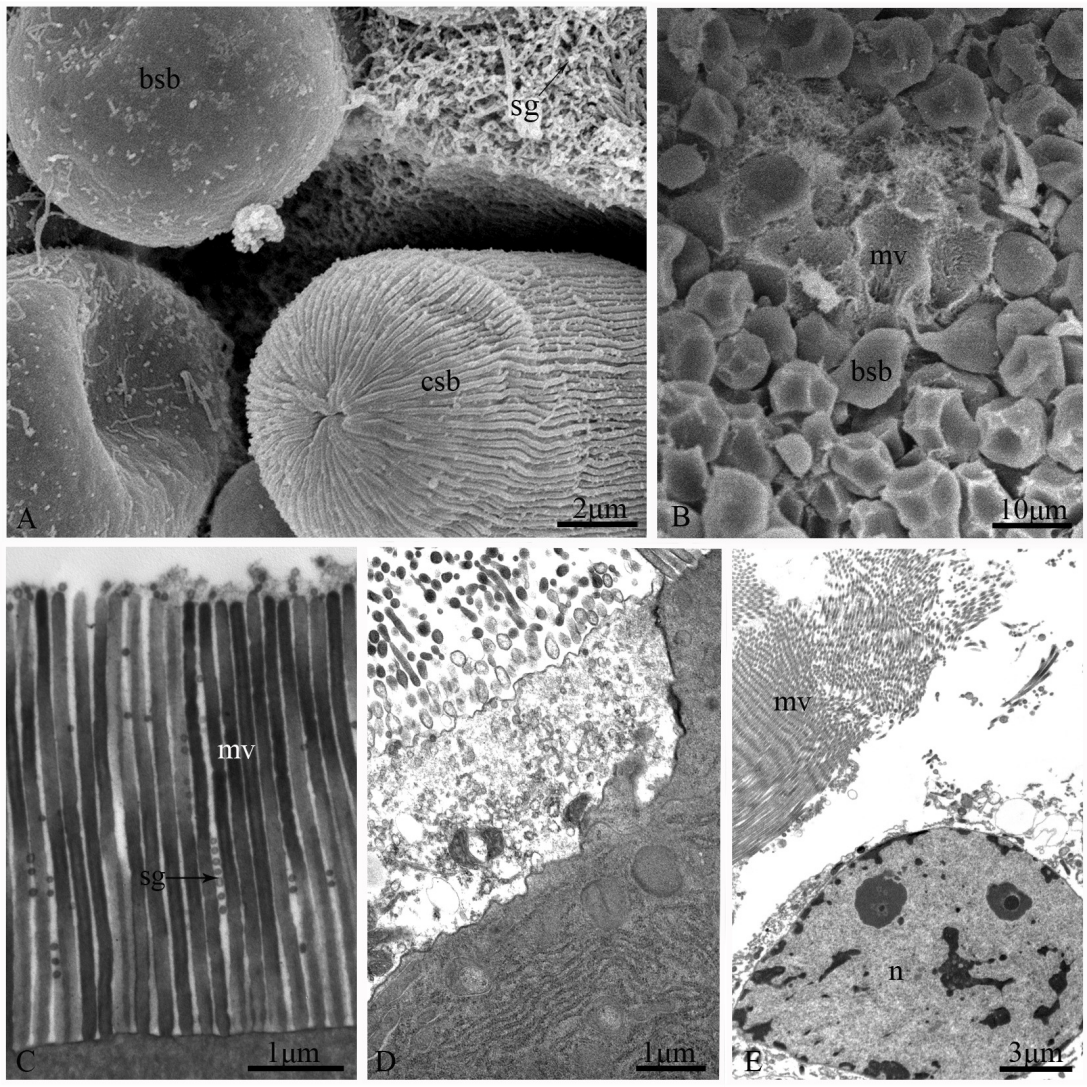
Fine structure of the secretory devices of the midgut of *G*. *gratiosa*. (A) Secretory devices in the gastric caeca, SEM. (B) Secretory devices in the ventriculus, SEM. (C) Merocrine secretion, TEM. (D) Apocrine secretion, TEM. (E) Holocrine secretion, TEM. bsb: bulb-shaped bubble; csb: cylindrical-shaped bubble; sg: secretory granule; mv: microvilli; n: nucleus. Scale bars: (A) = 2 μm; (B) = 10 μm; (C and D) = 1 μm; (E) = 3 μm.

Two secretory devices, merocrine and apocrine, were found in the apical membrane of principal cells (Fig 8A–8D). The merocrine devices usually bud to form microvilli and release secretions into the gut lumen (Fig 8C). The apocrine devices have two different shapes: (1) a bulb-shaped bubble with a smooth surface (Fig 8A and 8B), which is the most common shape among insects; and (2) a cylindrical-shaped apocrine bubble (Fig 8A) with the membrane surface developing regular longitudinal ridges (Fig 8A), which primarily occurs in the portion with the gastric caeca. However, secretion from both merocrine and bulb-shaped apocrine devices was observed along the entire midgut. In addition, holocrine secretion was observed by TEM in the gastric caeca of the midgut (Fig 8E).

## Discussion

Gastric caeca occurring in the midgut of many insects vary among taxa with regard to shape, number, size and position [2]. In Orthoptera, the caeca usually originate at the anterior end of the midgut. In Gryllidae, the two caeca are bulbous in shape [5, 14], whereas six bi-lobed finger-shaped caeca occur in Caelifera [2]. The midgut of *G*. *gratiosa* consists of two bulbous gastric caeca and a cylindrical ventriculus. The gastric caeca originate at the anterior end of the ventriculus, similar to other members of Ensifera, e.g., *Melanogryllus desertus* (Orthoptera: Gryllidae) [14], *Mecopoda niponensis* (Orthoptera: Mecopodidae) [9], and *Gryllodes sigillatus* (Orthoptera: Gryllidae) [5]. Epithelial folds can increase the area of the midgut and are commonly observed in the gastric caeca. In *G*. *gratiosa*, epithelial folds were observed at the distal end of both gastric caeca, similar to those in *G*. *sigillatus* [5] and *M*. *niponensis* [9]. In grasshoppers, the epithelium of the caeca is folded into longitudinal ridges [2, 10–12].

The epithelium of most insect midguts contains four cell types: principal, goblet, regenerative and endocrine [2]. Goblet cells are only found in the midguts of some insects, e.g., Ephemeroptera and Lepidoptera [15]. Three midgut epithelial cell types, principal, regenerative and endocrine cells, were observed in *G*. *gratiosa*, similar to previous reports for other Orthoptera members [5, 12, 14, 16]. Endocrine cells in insect midgut perform important roles by releasing various peptides in many key physiological functions, and the kind and distribution of regulatory peptides vary with different species [17]. Thus, further research in this area would be needed.

Principal cells exhibit the cellular structures of classic secretory cells: microvilli, large nuclei, mitochondria, ribosomes, RER, Golgi complex, and vesicles [18]. Differences in the regional ultrastructure of principal cells were observed along the midgut of *G*. *gratiosa*, a phenomenon also observed in other insects [12, 19, 20]. Regional differences in the principal cells of *G*. *gratiosa* are highlighted by the length of microvilli and the number and distribution of lipid droplets, lysosomes, MVBs, autophagosomes and cylindrical-shaped apocrine secretions.

Microvilli dramatically increase the apical membrane area of a cell for enzyme secretion and absorption of digested products, and in *G*. *gratiosa*, longer microvilli were found in the posterior midgut than in the other three regions of the midgut. Therefore, high rates of absorption likely occur in the posterior midgut.

Lipids play an important role in insects for energy storage for survival, development and subsequent reproduction [2]. Neutral lipids, digested and absorbed from food, consist predominantly of triacylglycerols or cholesteryl esters, and the core of lipid droplets is surrounded by a monolayer of phospholipids and associated proteins [21, 22]. Lipid droplets are present in various cell types of all organisms, from prokaryotes to eukaryotes [16, 22, 23]. Our study revealed the highest concentration of lipid droplets in the gastric caeca of *G*. *gratiosa*. Therefore, gastric caeca are likely a vital part of the midgut involved in lipid accumulation, as observed in *Abracris flavolineata* (Orthoptera: Acrididae) [2] and *Brontocoris tabidus* (Heteroptera: Pentatomidae) [24].

The intracellular storage and use of lipids are critical for maintaining cellular energy homeostasis. Lysosomes, a type of membrane-bound organelle [25], perform multiple functions in addition to degradation, including energy metabolism, secretion and plasma membrane repair [26]. MVBs and autophagosomes are closely related to lysosomes in the cellular energy balance. MVBs are a special type of late endosome that primarily separates and delivers proteins to lysosomes for degradation (Fig 4A and 4C) [27]. The proteins separated and delivered by MVBs vary for different cell types; for example, in mammotrophic hormone-producing cells of the rat anterior pituitary gland, MVBs regulate the secretory process by overproducing secretory granules [28]. During autophagy, cellular components are degraded in lysosomes (Fig 4D), assuring the removal of altered or dysfunctional proteins and organelles [29]. Recent studies reveal that multiple mechanisms operate in the delivery of functional proteins and organelles to lysosomes [30]. One such mechanism is fusion of autophagosomes with MVBs to generate an amphisome, which fuses with the lysosome to degrade the material contained within [31]. Additionally, autophagosomes have a key role in lipid metabolism by shuttling lipid droplets to lysosomes (Fig 4B), where they are hydrolyzed into free fatty acids and glycerol [29, 32]. MVBs, lysosomes and autophagosomes and lipid droplets were found together in the principal cells of the gastric caeca in *G*. *gratiosa*, similar to the mammotrophic hormone-producing cells of rat [28]. Therefore, we propose the existence of a regulatory mechanism of lysosomes over secretory granules, with this mechanism being associated with intracellular lipid metabolism in *G*. *gratiosa*.

Principal cells, particularly those in the gastric caeca, show intense secretory activity, and their cytoplasm contains abundant organelles related to secretion, such as RER, Golgi and secretory vesicles [18]. Three mechanisms release the products of secretion, most likely including digestive enzymes and other proteins, into the midgut lumen [33]: (a) holocrine, (b) merocrine and (c) apocrine. During holocrine secretion, all secretory vesicles and the cytoplasm are released by rupture of the plasma membrane and cellular degradation. In merocrine secretion, or exocytosis, the products in secretory vesicles reach the lumen via fusion of the limiting membrane of a vesicle with the apical membrane, without cytoplasm loss. In contrast, the mechanism for apocrine secretion involves loss of part of the apical cytoplasm as secretory vesicles are released. In *G*. *gratiosa*, a distinctive type of apocrine bleb was found in the gastric caeca (Fig 8A), with morphology that differed from that of the common apocrine bleb (Fig 8A and 8B). Further studies are required to ascertain whether these two types of apocrine blebs have different contents and functions.

In the present study, the morphology and structure of the midgut of *G*. *gratiosa* were examined. The results showed that the midgut of *G*. *gratiosa* consists of two bulbous gastric caeca and a tubular ventriculus. The organizational structure of these two components is similar. Regional ultrastructural differences along the entire midgut were observed for principal cells, such as in the gastric caeca, with abundant lysosomes, MVBs, autophagosomes, Golgi, lipid droplets and a type of distinctive apocrine bleb. These structural differences suggest that the function of the gastric caeca is more similar to that of a specialized gland. Therefore, we suggest that the gastric caeca play an essential role in functions of secretion and energy storage, whereas an absorptive function may be more important for the ventriculus.

## References

[1] Landim C, Da C, Moraes De R. Aspectos da ultra-estrutura dos cecos gástricos da larva de *Odontosciara sp*. (Diptera: Sciaridae). Zoologia. 1985; 3(4):181–8.

[2] Chapman R. Alimentary canal, digestion and absorption. Cambridge University Press 1998 doi: 10.1017/CBO9780511818202

[3] Terra WR, Espinoza-Fuentes FP, Ribeiro AF, Ferreira C. The larval midgut of the house fly (*Musca domestica*): ultrastructure, fluid fluxes and ion secretion in relation to the organization of digestion. Journal of Insect Physiology. 1988; 34(6):463–72. doi: 10.1016/0022-1910(88)90187-4

[4] Rost-Roszkowska MM, Piłka M, Szymska R, Klag J. Ultrastructural studies of midgut epithelium formation in *Lepisma saccharina L*. (Insecta, Zygentoma). Journal of Morphology. 2007; 268(3):224–31. doi: 10.1002/jmor.10513 17286269 10.1002/jmor.10513

[5] Biagio FP, Tamaki FK, Terra WR, Ribeiro AF. Digestive morphophysiology of *Gryllodes sigillatus* (Orthoptera: Gryllidae). Journal of Insect Physiology. 2009; 55(12):1125–33. doi: 10.1016/j.jinsphys.2009.08.015 19715697 10.1016/j.jinsphys.2009.08.015

[6] Nardi JB, Miller LA, Bee CM, Jr LR, Denlinger DL. The larval alimentary canal of the Antarctic insect, *Belgica antarctica*. Arthropod Structure & Development. 2009; 38(5):377–89. doi: 10.1016/j.asd.2009.04.003 19401239 10.1016/j.asd.2009.04.003

[7] Zhang FM, Zhang CN, Dai W, Zhang YL. Morphology and histology of the digestive system of the vector leafhopper *Psammotettix striatus* (L.) (Hemiptera: Cicadellidae). Micron. 2012; 43(6):725–38. doi: 10.1016/j.micron.2012.01.004 22341338 10.1016/j.micron.2012.01.004

[8] Terra WR. Evolution of digestive systems in insects. Annual Review of Entomology. 1990; 35:181–200. doi: 10.1146/annurev.en.35.010190.001145

[9] Li ZY, Zheng ZM. Fine structure of the alimentary canals of *Mecopoda niponensi*. Entomological Knowledge. 2004; 41(3):244–9.

[10] Droste HJ, Zebe E. Resorption und Stoffwechsel von Glucose im Darmtrakt der Wanderheuschrecke *Locusta migratoria*. Journal of Insect Physiology. 1974; 20(12):2385–96. doi: 10.1016/0022-1910(74)90025-0 4436583 10.1016/0022-1910(74)90025-0

[11] Anstee JH, Charnley AK. Effects of frontal ganglion removal and starvation on activity and distribution of six gut enzymes in Locusta. Journal of Insect Physiology. 1977; 23(8):965–74. doi: 10.1016/0022-1910(77)90124-X

[12] Marana SR, Ribeiro AF, Terra WR, Ferreira C. Ultrastructure and secretory activity of *abracris flavolineata* (Orthoptera: Acrididae) midguts. Journal of Insect Physiology. 1997; 43(5):465–73. doi: 10.1016/S0022-1910(96)00117-5

[13] Li K, Yang YJ, Mao L, Zhang JH. Morphology and ultrastructure of the spermatheca of *Pararcyptera microptera meridionalis* (Orthoptera: Arcypterinae). Acta Entomologica Sinica. 2016 (5):523–9. doi: 10.16380/j.kcxb.2016.05.006

[14] Cakici O, Ergen G. Histologic Description of Midgut in *Melanogryllus desertus* (Pallas, 1771) (Orthoptera: Gryllidae). Biharean Biologist. 2012; 6(2):108–11.

[15] Happ GM. Insect Ultrastructure: Plenum Press; 1984 doi: 10.2307/1309435

[16] Wältermann M, Steinbüchel A. Neutral lipid bodies in prokaryotes: recent insights into structure, formation, and relationship to eukaryotic lipid depots. Journal of Bacteriology. 2005; 187(11):3607–19. doi: 10.1128/JB.187.11.3607-3619.2005 15901682 10.1128/JB.187.11.3607-3619.2005 PMC1112053

[17] Wegener C and Veenstra JA. Chemical identity, function and regulation of enteroendocrine peptides in insects. Current Opinion in Insect Science. 2015; 11:8–13. doi: 10.1016/j.cois.2015.07.003 28285763 10.1016/j.cois.2015.07.003

[18] Rothman JE, Orci L. Molecular dissection of the secretory pathway. Nature. 1992; 355(6359):409 doi: 10.1038/355409a0 1734280 10.1038/355409a0

[19] Wanderley-Teixeira V, Teixeira AAC, Cunha FM, Costa MK, Veiga AF, Oliveira JV. Histological description of the midgut and the pyloric valve of *Tropidacris collaris* (stoll, 1813) (*Orthopetera*: *Romaleidae*). Brazilian Journal of Biology. 2006; 66(4):1045–9. doi: 10.1590/S1519-69842006000600011 10.1590/s1519-69842006000600011 17299940

[20] Roelfstra L, Vlimant M, Betschart B, Pfister K, Diehl PA. Light and electron microscopy studies of the midgut and salivary glands of second and third instars of the horse stomach bot, *Gasterophilus intestinalis*. Medical & Veterinary Entomology. 2010; 24(3):236 doi: 10.1111/j.1365-2915.2010.00881.x 20534009 10.1111/j.1365-2915.2010.00881.x

[21] Martin S, Parton RG. Caveolin, cholesterol, and lipid bodies. Seminars in Cell & Developmental Biology. 2005; 16(2):163 doi: 10.1016/j.semcdb.2005.01.007 15797827 10.1016/j.semcdb.2005.01.007

[22] Martin S, Parton RG. Lipid droplets: a unified view of a dynamic organelle. Nature Reviews Molecular Cell Biology. 2006; 7(5):373–8. doi: 10.1038/nrm1912 16550215 10.1038/nrm1912

[23] Murphy DJ. The biogenesis and functions of lipid bodies in animals, plants and microorganisms. Progress in Lipid Research. 2001; 40(5):325–438. doi: 10.1016/S0163-7827(01)00013-3 11470496 10.1016/s0163-7827(01)00013-3

[24] Mariadocarmoq F, Zanuncio Z, Clóvisa N, Franciscos R, Joséeduardo S. Ultrastructure of the Digestive Cells in the Midgut of the Predator *Brontocoris Tabidus* (Heteroptera: Pentatomidae) After Different Feeding Periods on Prey and Plants. Annals of the Entomological Society of America. 2009; 102(1):119–27. doi: 10.1603/008.102.0113

[25] Xu H, Ren D. Lysosomal Physiology. Annual Review of Physiology. 2015; 77(1):57 doi: 10.1146/annurev-physiol-021014-071649 25668017 10.1146/annurev-physiol-021014-071649 PMC4524569

[26] Settembre C, Fraldi A, Medina DL, Ballabio A. Signals from the lysosome: a control centre for cellular clearance and energy metabolism. Nature Reviews Molecular Cell Biology. 2013; 14(5):283 doi: 10.1038/nrm3565 23609508 10.1038/nrm3565 PMC4387238

[27] Piper RC, Katzmann DJ. Biogenesis and Function of Multivesicular Bodies. Annual Review of Cell & Developmental Biology. 2007; 23(1):519–47. doi: 10.1146/annurev.cellbio.23.090506.123319 17506697 10.1146/annurev.cellbio.23.090506.123319 PMC2911632

[28] Smith RE, Farquhar MG. Lysosome Function in the Regulation of the Secretory Process in Cells of the Anterior Pituitary Gland. Journal of Cell Biology. 1966; 31(2):319 19866704 10.1083/jcb.31.2.319 PMC2107048

[29] Singh R, Cuervo Maria A. Autophagy in the Cellular Energetic Balance. Cell Metabolism. 2011; 13(5):495–504. doi: 10.1016/j.cmet.2011.04.004 21531332 10.1016/j.cmet.2011.04.004 PMC3099265

[30] Kadandale P, Kiger AA. Role of selective autophagy in cellular remodeling: "self-eating" into shape. Autophagy. 2010; 6(8):1194–5. doi: 10.1073/pnas.0914168107 Epub 2010 Nov 16. 20890115 10.4161/auto.6.8.13476 PMC3359492

[31] Fader CM, Colombo MI. Autophagy and multivesicular bodies: two closely related partners. Cell Death & Differentiation. 2009; 16(1):70–8. doi: 10.1038/cdd.2008.168 Epub 2008 Nov 14. 19008921 10.1038/cdd.2008.168

[32] Singh R, Kaushik S, Wang Y, Xiang Y, Novak I, Komatsu M, et al Autophagy regulates lipid metabolism. Nature. 2009; 458(7242):1131–5. doi: 10.1038/nature07976 19339967 10.1038/nature07976 PMC2676208

[33] Terra WR, Ferreira C. Comprehensive Molecular Insect Science In: Gilbert LI, Iatrov K, Gill SS, editors. Biochemistry of Digestion. 4: Oxford; 2005 p. 171–224.

